# CAKUT: A Pediatric and Evolutionary Perspective on the Leading Cause of CKD in Childhood

**DOI:** 10.3390/pediatric15010012

**Published:** 2023-02-10

**Authors:** Robert L. Chevalier

**Affiliations:** Department of Pediatrics, The University of Virginia, Charlottesville, VA 22908, USA; rlc2m@virginia.edu

**Keywords:** congenital anomalies of the kidney and urinary tract (CAKUT), chronic kidney disease, pediatrics, evolution

## Abstract

The global prevalence of chronic kidney disease (CKD) is increasing rapidly, due to increasing environmental stressors through the life cycle. Congenital anomalies of kidney and urinary tract (CAKUT) account for most CKD in children, with a spectrum that can lead to kidney failure from early postnatal to late adult life. A stressed fetal environment can impair nephrogenesis, now recognized as a significant risk factor for the development of adult CKD. Congenital urinary tract obstruction is the leading cause of CKD due to CAKUT and can itself impair nephrogenesis as well as contribute to progressive nephron injury. Early diagnosis by ultrasonography in fetal life by an obstetrician/perinatologist can provide important information for guiding prognosis and future management. This review focuses on the critical role played by the pediatrician in providing timely evaluation and management of the patient from the moment of birth to the transfer to adult care. In addition to genetic factors, vulnerability of the kidney to CKD is a consequence of evolved modulation of nephron number in response to maternal signaling as well as to susceptibility of the nephron to hypoxic and oxidative injury. Future advances in the management of CAKUT will depend on improved biomarkers and imaging techniques.

## 1. Chronic Kidney Disease over the Life Span

**Figure 1:**
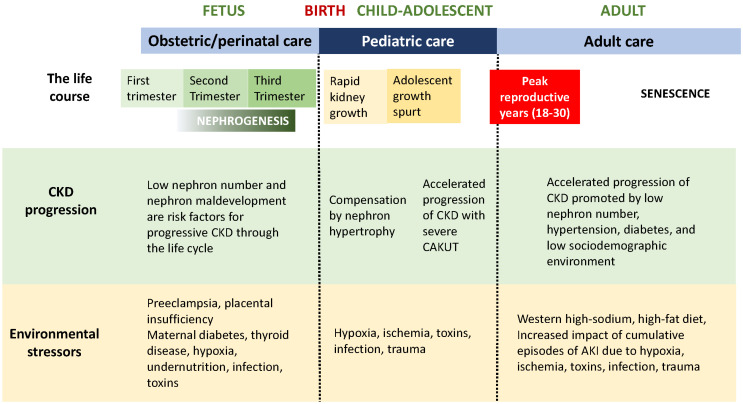
Life course of the CAKUT patient: Chronic kidney disease progression and environmental stressors.

The global prevalence of chronic kidney disease (CKD) is increasing rapidly and is often detected only after a significant loss of function [1]. Epidemiologic studies have revealed that progression of CKD results from the complex interactions of genetic, epigenetic, and the cumulative impact of environmental factors over the life span (Figure 1) [2]. Following the critical discoveries of David Barker and associates that adult cardiovascular disease is linked to low birth weight, the discipline of developmental origins of health and disease (DOHaD) is now firmly established [3]. Importantly, the maternal-fetal and early postnatal environment are now recognized to contribute to the progression of chronic adult disorders including hypertension, diabetes, and CKD (Figure 1) [4]. The prevalence of CKD in the global population increases dramatically in adults over 40 years of age, initially well below 1% in children, rising to greater than 40% in late adulthood [1]. Late progression of CKD was revealed in a study of military recruits with a history of kidney disease in childhood. Even though kidney function was normal in these patients at 18 years of age, they revealed an increased risk of end-stage kidney disease later in adulthood [5]. The present review emphasizes the important role played by the pediatrician, pediatric nephrologist, and pediatric urologist in the long-term health outcome of infants born with reduced nephron number or congenital anomalies of the kidney and urinary tract (CAKUT), both representing a continuum of nephrogenesis under stress [6]. Positioned in health care delivery over the crucial interval between an obstetrician/perinatologist and adult health care provider (internist, nephrologist, and urologist), the pediatrician’s responsibility is to optimize kidney health of the child with CAKUT through the period of the greatest somatic growth (Figure 1).

The pediatrician will most often become aware of the diagnosis of CAKUT in a patient through information transmitted by the mother’s obstetrician, who will have made a tentative diagnosis from screening ultrasound examinations. As outlined in the figures, the broad category of CAKUT has its origins in fetal life, with its progression and outcome determined by the maldevelopment of specific components of the kidneys and urinary tract. Thus, the spectrum of disease in the individual patient can range from a transient sign (such as unilateral mild hydronephrosis) to absence of both kidneys and early neonatal death. The preterm infant or the child with intrauterine growth restriction is at risk for the development of a reduced nephron number, and therefore, requiring careful monitoring throughout childhood. A baby that fails to urinate in the first 24 h of life or who manifests any congenital malformation in the postnatal examination is at increased risk for CAKUT and should undergo abdominal ultrasonography. The discovery of any abnormalities should then prompt the involvement of a pediatric nephrologist and/or pediatric urologist to plan a course of investigation and treatment, as recommended in this review.

## 2. Nephrogenesis: Determination of Nephron Number

**Figure 2:**
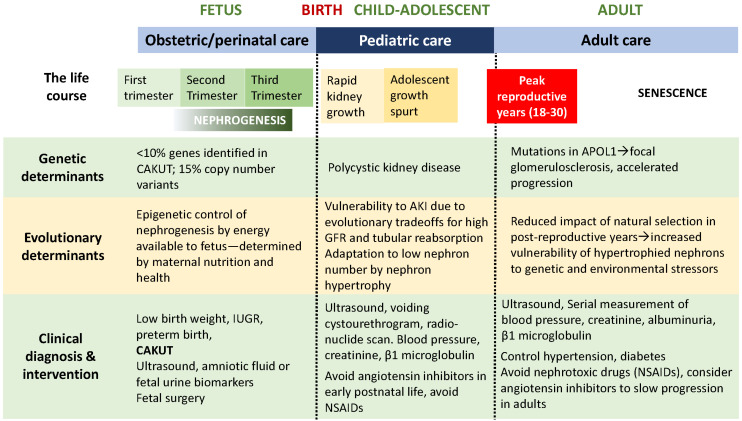
Life course of the CAKUT patient: Genetic determinants, evolutionary determinants, clinical diagnosis and intervention.

Kidney health is the product of the number of functioning nephrons through the life course, which begin their development in the first trimester of gestation. Genetic and epigenetic factors play a major role in early development, with severe organ maldevelopment leading to spontaneous abortion that is often undetected in the first weeks of embryonic life (Figure 2). Unlike most other organs, the period of nephron formation is prolonged, beginning at the 10th week and complete by the 36th week of gestation, with 90% of nephrons developing during the third trimester (Figure 1 and Figure 2). Thus, stresses to the fetal environment, particularly through later pregnancy, have a major impact on the number of nephrons at birth. This number can vary from 200,000 to 2 million nephrons per kidney, with nephron number being correlated with birth weight [7]. Low birth weight can result from intrauterine growth restriction (IUGR) or preterm birth, both of which are associated with low nephron number, which in turn increase susceptibility to CKD in adulthood [4,8]. A history of maternal malnutrition, diabetes, nephrotoxic medications, or preeclampsia, therefore, predisposes the fetus to a reduced nephron number (Figure 1).

## 3. CAKUT and Pediatric CKD

While contributing over 50% of pediatric CKD cases, the prevalence of CAKUT is very low (<1% of live births) compared to low birth weight (15% of live births) [9]. The wide variation of CAKUT results in a spectrum of compromised kidney function ranging from mild to severe. It is important to consider maldevelopment of the collecting system as well as the kidneys because their functional interaction during fetal life determines the severity of disease and rate of CKD progression. Thus, distinguishing the upper urinary tract (kidneys and ureters) from the lower tract (bladder and urethra) and unilateral vs. bilateral kidney anomalies are important determinants of the clinical outcome.

## 4. Genetics of CAKUT

An inherited etiology has been estimated to be responsible for up to 20% of CAKUT cases [10]. A number of genes have been identified as responsible for both syndromic and nonsyndromic CAKUT: single gene mutations account for 10% of sporadic cases, and more recently, an additional 15% were discovered to be associated with copy number variants [11,12]. This leaves 75% of cases without current genetically determined etiology. Thus, every patient should undergo a thorough review of family history for the presence of CAKUT, and be referred to genetic counseling when indicated [13,14]. Recent studies reveal that epigenetic regulation of nephron differentiation by histone modifications and DNA methyltransferases regulate nephron number in response to energy provided to the fetus through the placenta [15]. By increasing susceptibility to glomerular injury, APOL1 mutations, present in some populations of African ancestry, accelerate the rate of CKD progression in adulthood (Figure 2) [16].

## 5. Animal Models of Congenital Urinary Tract Obstruction

Although obstructive nephropathy is the leading cause of CAKUT, congenital urinary tract obstruction as a clinical entity is difficult to define because of the complexity of the anatomy and physiology of the fetal urinary tract subjected to maldevelopment. Craig Peters defined it as “a condition of impaired urinary drainage which, if uncorrected, will limit the ultimate functional potential of a developing kidney” [17]. The challenge is to determine the mechanisms responsible for nephron injury resulting from urinary tract obstruction during and after the completion of nephrogenesis. Experimental studies have revealed some of these mechanisms in the developing kidney, and the importance of timing on surgical correction. Kidney growth in the neonatal rat subjected to unilateral partial unilateral ureteral obstruction (UUO) is impaired at a critical degree of ureteral stenosis (approximately 70% reduction in ureteral diameter), whereas kidney growth is preserved with less severe obstruction [18]. Notably, signs of nephron injury (tubular atrophy and interstitial fibrosis), reduced nephron number, and reduced glomerular filtration rate can develop before pelvic dilatation and renal growth impairment are detected [18]. Release of partial UUO in the neonatal mouse can result in the arrest of progressive nephron loss and resolution of tubular atrophy and interstitial fibrosis [19]. Whereas chronic partial UUO impairs growth of the obstructed kidney, compensatory growth is induced in the contralateral kidney. Early release of the obstruction normalizes growth of both kidneys, whose weight is not different from that of sham-operated animals [19]. Because CAKUT often occurs in low-birth weight infants (born with low nephron number), the results of partial UUO in wild-type mice were compared with those in mutant mice with a 50% reduction in nephron number [20]. The mouse used in these experiments is the Oligosyndactylism mouse, a radiation-induced mutation on mouse chromosome 8 associated with early postimplantation lethality in homozygotes and abnormal development of the limbs and a 50% reduction in nephrons in kidneys of heterozygotes.

Partial UUO caused further nephron loss, and release of obstruction failed to reverse kidney injury in mice with reduced nephron number, indicating that preterm infants with CAKUT may be at increased risk for progressive CKD [20].

## 6. Obstetrics and Fetal Management of CAKUT

The most severe forms of CAKUT develop in the first trimester, and an initial screening antenatal ultrasound can be performed by the obstetrician/perinatologist between 16 and 20 weeks of gestation, when the urine-filled bladder is detectable. Early anomalies include bilateral renal agenesis, severe forms of bilateral renal dysplasia, vesicoureteral reflux, and congenital lower urinary tract obstruction (Table 1 and Table 2) [6]. Dilatation of the renal pelvis (hydronephrosis) can result from vesicoureteral reflux as well as from urinary tract obstruction, and reflux nephropathy is a significant cause of CAKUT that is complicated by postnatal urinary tract infection [21]. Obstruction can develop at the ureteropelvic junction (the most common upper urinary tract obstruction) [22], ureterovesical junction, or urethra (Table 2). In the case of posterior urethral valves, nephron dysplasia develops in the third trimester due to increased hydrostatic pressure in the collecting system as well as ongoing injury to intact nephrons caused by abnormal ureteral and bladder function through the second trimester. Prenatal surgical intervention to repair the lesion or divert the obstruction with a shunt has met with limited success, and surviving infants develop early CKD with progression to kidney failure in the most severe cases [23]. 

The consequences of unilateral anomalies, such as unilateral ureteropelvic junction obstruction or vesicoureteral reflux, are generally less severe than those resulting from bilateral anomalies. Complete ureteral obstruction results in severe kidney maldevelopment, exemplified by the nonfunctioning multicystic-dysplastic kidney that is often associated with ureteral atresia. However, multicystic-dysplastic kidney is often associated with abnormalities of the contralateral kidney, such as ureteropelvic junction obstruction, which may require early postnatal intervention [24]. Either autosomal recessive or autosomal dominant polycystic kidney disease can present in the fetus, and involvement of the two kidneys can be asymmetrical. Diagnosis can be made by postnatal ultrasound combined with family history and genetic testing [25]. 

Because most nephrons are formed in the third trimester, kidney growth can be monitored through the second half of pregnancy by serial ultrasound studies. Many cases of apparent mild pelvic dilatation resolve with progression of pregnancy, underscoring the importance of serial antenatal sonography. This will reveal whether one or both kidneys are affected and whether the bladder and ureters also become dilated. The development of cystic kidney disease, inadequate kidney growth (hypoplasia), or compensatory renal hypertrophy in response to an absent or poorly functioning contralateral kidney are also detectable (Table 1).

Amniotic fluid volume is of major significance, most of which is composed of fetal urine output in second and third trimesters, thereby providing a measurement of fetal kidney function. Importantly, normal fetal pulmonary development is dependent upon adequate contribution of urine to the amniotic fluid that is swallowed by the fetus. Oligohydramnios can therefore result from bilateral renal agenesis (fetal anuria) or bilateral hypoplasia/dysplasia, cystic kidney disease, or lower urinary tract obstruction (Table 1). The result of severe fetal oliguria is progression of the Potter sequence, which results in a phenotype with characteristic compressed facies, wrinkled skin, and pulmonary hypoplasia, which can lead to progressive pulmonary disease or early postnatal death [26]. The presence of oligohydramnios is, therefore, an ominous sign that must be pursued with ultrasonography to rule out bilateral upper tract or lower urinary tract obstruction that is impairing fetal urine flow.

## 7. Postnatal Management by a Pediatrician, Pediatric Nephrologist, and Urologist

For infants born with a diagnosis of suspected CAKUT based on antenatal sonography, postnatal ultrasound should be obtained expeditiously if the patient has not voided within the first 24 h [27]. For unilateral hydronephrosis in otherwise healthy infants, the first postnatal ultrasound should be obtained after the first 48 h of life, to allow for increasing postnatal kidney function within the first week of life, and the severity of reflux/obstruction can be gauged by the degree of pelvic and calyceal dilatation. Inspection of ureters and bladder by ultrasonography may suggest the location of the lesion (Table 2). Voiding cystourethrogram can be obtained to rule out vesicoureteral reflux, followed by radionuclide MAG-3 scan to rule out obstruction [28]. If hydronephrosis is mild, follow-up sonograms can be performed to detect progressive hydronephrosis requiring pyeloplasty vs. spontaneous resolution of ureteropelvic junction obstruction [22]. The argument for early surgical intervention is based on the risk of continued nephron injury with prolonged exposure to increased intrapelvic pressure, as demonstrated in experimental models [18].

Posterior urethral valves represent the most common cause of lower urinary tract obstruction, with bilateral hydronephrosis, increased renal echogenicity (due to dysplasia), bladder dilatation, and thickening (Table 3). The etiology is unknown, with a broad spectrum of severity [23]. Fetal karyotyping can rule out chromosomal abnormality, and serial fetal urine sodium and chloride concentration >90 mmol/L and urine osmolality <210 mmol/kg are associated with severe fetal renal functional impairment [29]. For affected fetuses not undergoing prenatal intervention, prompt postnatal ultrasound and voiding cystourethrogram should be performed, followed by bladder drainage or valve ablation. Once the patient is stabilized, radionuclide scan with MAG-3 can reveal the presence of upper tract obstruction, and a dimercaptosuccinic acid (DMSA) radionuclide scan can reveal differential function of the two kidneys and kidney scarring [28].

Following delivery of an infant not undergoing prenatal abdominal sonography, the first physical examination may reveal a palpable abdominal mass. Obstructive nephropathy is the most common etiology, followed by cystic kidney disease, renal vein thrombosis, hypertrophied solitary kidney, and malignancy. Evaluation should include imaging by ultrasonography followed by radionuclide scan and biopsy, if indicated [30]. 

Several syndromes diagnosed at birth include CAKUT and affected patients should be evaluated for possible urinary tract obstruction or reflux (Table 4). Conversely, all patients with CAKUT should be carefully examined for the presence of nonrenal anomalies as well as syndrome-specific signs and symptoms [13]. In addition to patients with features of syndromes evident on physical examination of the lower body, infants with congenital hypothyroidism have an odds ratio for CAKUT of 13 compared to the general population (Table 4) [31]. Malformations of the outer ear or presence of a single umbilical artery are associated with an increased prevalence of CAKUT. The spectrum of CAKUT prevalent in patients with chromosomal abnormalities is well established, with vesicoureteral reflux and lower urinary tract obstruction being predominant [10]. In addition to CAKUT, a recent report revealed that children with Down syndrome have smaller kidneys and decreased glomerular filtration rate compared to age-matched controls [32]. 

Care of the child will be determined by the nature and severity of the disorder: the spectrum of CAKUT is very broad. General pediatricians need to recognize the vulnerability of kidneys to injury in low-birth weight infants, particularly patients discharged from a neonatal intensive care unit during which they were exposed to hypoxia and nephrotoxic drugs [33]. Because of the increased risk for progressive CKD in the patient with low nephron endowment, all infants with low birth weight, preterm birth, and/or intrauterine growth restriction should have regular blood pressure measurement and periodic urinalysis (Figure 2) [34]. Periodic ultrasonography should be considered in all patients with CAKUT to document normal and/or compensatory kidney growth, as well as urinalysis and plasma creatinine concentration. Patients with lower urinary tract anomalies may require monitoring for urinary tract infection. Avoidance of nephrotoxic drugs, including nonsteroidal anti-inflammatory drugs (NSAIDs), is important—especially in states of volume depletion. Administration of angiotensin converting enzyme inhibitors should be avoided because of the lack of effectiveness in slowing CKD in children with hypodysplasia [35]. For patients with reduced kidney function, there is increased risk for acceleration of CKD with the onset of the adolescent growth spurt (Figure 1), and episodes of acute kidney injury can accelerate even mild CKD in the patient with CAKUT [36]. 

The infant born with a single functioning kidney deserves special consideration. This category includes patients with unilateral renal agenesis and those with a normal kidney and contralateral nonfunctioning kidney (multicystic-dysplastic, severe obstructive nephropathy, or tumor). Whereas compensatory growth of the intact kidney had long been considered adequate to maintain kidney function through the life span, more recent reports revealed the development of CKD in adulthood [37]. Significantly, infants with a small single functioning kidney are more likely to develop CKD in adulthood, whereas those developing compensatory kidney growth in utero have reduced risk of CKD in adulthood [38,39,40]. This underscores the importance of compensatory adaptation during the period of nephrogenesis. Although most are otherwise healthy through childhood, all children with single functioning kidney require regular monitoring of blood pressure, urinalysis, plasma creatinine concentration, and kidney growth. 

## 8. Transfer to Adult Care: Internist, Nephrologist, and Urologist

For the reasons discussed above, patients with CAKUT benefit from a continuum of care with close communication between teams of physicians responsible for each stage of the life course (Figure 1 and Figure 2). Whereas CAKUT comprises more than 50% of cases of CKD in pediatrics, of all the children with CAKUT progressing to end-stage kidney disease, over 50% will not require renal replacement therapy until the fourth decade of life [36]. Just as the general pediatrician must be made aware of the significance of nephron deficit in the low-birth weight patient, the physician to adults is responsible for an even greater span of the patient’s life and should also be aware of this. Moreover, exposure to a high-sodium, high-fat Western diet is contributing to the rise of hypertension, diabetes, and metabolic syndrome—all of which promote the acceleration of CKD [41]. Continued participation of the patient and family in understanding the importance of lifelong monitoring of kidney health is also a key factor in optimizing care.

## 9. Evolution of the Kidney: An Explanation for the Epidemiology of Progressive CKD

What are the factors that drive the 10-fold variation in nephron number at birth, with a significant fraction of infants born with low nephron number and CAKUT, and that also account for the increasing prevalence of CKD in adulthood? Developed over the past 30 years, the new discipline of evolutionary medicine brings together anatomy, pathophysiology, and evolutionary biology to seek the ultimate cause of chronic disease [42]. The central principle driving this approach to medicine is that human anatomy and physiology are the product of evolutionary adaptation to the environment—an environment that has changed dramatically over the 4.5 billion-year history of our planet. Our ancestors adapted from a marine environment to freshwater and terrestrial environments, requiring marked changes in the evolution of eukaryotes, symbiosis (acquisition of mitochondria), multicellularity, and endothermy [43]. These innovations led to evolution of the mammalian nephron, with its high-pressure, high-filtering glomerulus requiring an energy-consuming tubule to reclaim 99% of the filtrate and a hyperosmotic medulla to produce concentrated urine [44]. These adaptations enabled the evolution of *Homo sapiens* from its primate ancestors over a period of 2 million years, and the migration of our species from Africa across the entire planet in the past 70,000 years [45]. The trade-off for this spectacular feat is the increased vulnerability of the nephron to podocyte loss due to high pressure/flow glomerular filtration, oxidative injury to the tubule resulting from high mitochondrial metabolism, and susceptibility of the medulla to hypoxic injury in its hypoxic/hyperosmotic microenvironment [43]. 

Evolution is the product of adaptation to the external environment through natural selection driven not by longevity, but by reproductive fitness constrained by available energy. Because reproductive success in *Homo sapiens* was favored by prioritization of fetal brain over kidney development, reduced nephron number represents an adaptation to maternal stress that is balanced by compensatory nephron hypertrophy that can begin in fetal life [15]. If the nephron deficit is not severe, and compensatory hypertrophy by remaining nephrons can preserve homeostasis, this evolutionary strategy can maintain homeostasis through peak reproductive years, after which natural selection plays a diminishing role (Figure 2). Although compensatory hypertrophy is an effective adaptation, cellular hypertrophy increases vulnerability to environmental stressors that are cumulative through senescence, with increasing prevalence of CKD in later life, a period less responsive to natural selection [15,46].

## 10. Answering the Questions of the Family and Child with CAKUT

The diagnosis and management of the patient with CAKUT are some of the most challenging in the practice of medicine. The most difficult questions and decisions arise in the antenatal period. In most cases, the etiology of the developmental anomaly is unknown, and even in cases of identifiable genetic disorders, the risk for CKD or its rate of progression are unpredictable. Referral to a genetic counselor should be available [14]. For the fetus with major congenital anomalies or chromosomal abnormalities, the possibility of early CKD, kidney failure, or death must be discussed with family, and parents or guardians should communicate relevant information to the patient and siblings when of appropriate age. 

The medical team should be aware of the cultural or ethnic affiliation of the patient and family, and to balance this context with current evidence-based medical information. For infants with hydronephrosis, the questions of long-term observation vs. surgical intervention should be discussed in conjunction with the pediatric urology team. For infants with posterior urethral valves, the long-term outcome is highly dependent on the severity of obstruction and kidney function in the first year of life [23]. Families of low-birth weight infants, particularly those discharged from a neonatal intensive care unit, must be made aware of the silent nature of CKD progression and the importance of blood pressure monitoring, imaging, and laboratory follow-up.

## 11. The Future: Advances in the Diagnosis and Management of CAKUT

Our kidneys play a central role in maintaining homeostasis and allowing us to adapt to our environment through the entire life cycle (Figure 1 and Figure 2). Because their development extends through most of fetal life (compared to other organs completing morphogenesis in the first trimester), prenatal diagnosis and treatment of CAKUT present multiple challenges. Ultrasound is the primary imaging modality in early pregnancy, and additional information can be gained by magnetic resonance imaging (MRI) [47]. A recent study of fetuses with ultrasound diagnosis of CAKUT revealed that a combination of amniotic fluid peptides and thymosin-β4 predicted postnatal kidney function with high specificity [48]. This represents a significant advance over reliance on serial ultrasonography, and even fetal urine electrolytes, which have poor predictive value. 

The greatest challenge in postnatal management of the patient with CAKUT is determining prognosis and monitoring progression of CKD. A recent report concluded that whereas urine albumin/creatinine ratio is a poor index of declining kidney function in children with CAKUT, urine α1-microglobulin and β2-microglobulin-to-creatinine ratio offer superior sensitivity and specificity [49]. Determination of glomerular filtration rate, regardless of technique, cannot distinguish between the aggregate function of smaller nephrons and that of fewer larger nephrons. Progress is being made in the development of noninvasive in vivo measurement of nephron number and size using cationized ferritin-enhanced magnetic resonance imaging (MRI) [50]. This technique can reveal changes in glomerular and tubular pathologic changes as well as hypertrophic growth [51]. The most difficult barrier remains the spontaneous termination of nephrogenesis by the 36th week of gestation, the ultimate goal being the provision of additional functioning nephrons. This is of particular concern in the very low birth weight infant born with incomplete nephrogenesis [52]. Unlike the ability of ectothermic vertebrates such as fish, which can adapt to nephron loss by regenerating nephrons in adulthood, endothermic mammals have evolved nephron hypertrophy, which provides limited adaptation through late adulthood [53]. Rapid advances in regeneration medicine bring hope to current renal replacement therapy options with biologic solutions that could vastly improve the quality of life or our patients with severe CAKUT [54].

## Figures and Tables

**Table 1:** Differential diagnosis of CAKUT.

Abnormal Collecting System (Duplicated, Refluxing, Obstructed)
Abnormal number or position of kidneys (ectopic, supernumerary, horseshoe kidney; renal agenesis)
Renal hypoplasia/dysplasia (with or without associated extrarenal anomalies)
Multicystic/polycystic kidney disease (unilateral or bilateral)
Oligohydramnios: Potter sequence (pulmonary hypoplasia with bilateral renal agenesis, severe posterior urethral valves, bilateral hypoplasia/dysplasia, or cystic kidney disease)
Rapid kidney growth (compensatory growth with decreased contralateral kidney function)

**Table 2:** Levels of congenital urinary tract obstruction.

**Upper Tract**
Ureteropelvic junction obstruction
**Lower tract**
Vesicoureteral obstruction, ureterocele
Posterior urethral valves, anterior urethral valves, urethral atresia

**Table 3:** Evaluation of lower urinary tract obstruction.

**Prenatal**
Fetal ultrasound: rule out decreased amniotic fluid volume, increased renal echogenicity, hydronephrosis, bladder dilatation
Fetal karyotyping: rule out chromosomal anomaly
Serial fetal urine sampling (oligohydramnios or bilateral hydronephrosis): rule out critical obstruction and consider prenatal surgery
**Postnatal**
Complete physical examination: rule out extrarenal anomalies
Postnatal ultrasound: rule out bladder thickening/trabeculation

**Table 4:** Renal hypoplasia/dysplasia with additional anomalies.

Prune-belly syndrome (Eagle-Barrett syndrome) Wrinkled abdominal skin Undeveloped abdominal musculature Undescended testes CAKUT (urethral hypoplasia, hydroureteronephrosis, dilated trabeculated bladder)
VATER, VACTRL syndrome Vertebral defects Anal atresia Cardiac defects Tracheo-esophageal fistula Limb abnormalities CAKUT (vesicoureteral reflux, unilateral renal agenesis, multicystic-dysplastic kidney, duplicated collecting system)
Bladder exstrophy Epispadias Pelvic bone anomalies CAKUT (unilateral renal agenesis, malrotated/ectopic kidney)
Myelomeningocele Neurogenic bladder Vesicoureteral reflux Urinary tract infection
Sacrococcygeal teratoma Bladder outlet obstruction hydronephrosis
Congenital hypothyroidism hypospadias hydronephrosis renal agenesis
Presence of outer ear abnormalities or single umbilical artery CAKUT (vesicoureteral reflux)
Chromosomal anomalies Down syndrome Turner syndrome
